# Variation in the Main Health-Promoting Compounds and Antioxidant Capacity of Three Leafy Vegetables in Southwest China

**DOI:** 10.3390/molecules28124780

**Published:** 2023-06-15

**Authors:** Yi Zhang, Wenli Huang, Chenlu Zhang, Huanhuan Huang, Shihan Yang, Yiqing Wang, Zhi Huang, Yi Tang, Xiaomei Li, Huashan Lian, Huanxiu Li, Fen Zhang, Bo Sun

**Affiliations:** 1 College of Horticulture, Sichuan Agricultural University, Chengdu 611130, China; 202001780@stu.sicau.edu.cn (Y.Z.); 2021305054@stu.sicau.edu.cn (W.H.); 2022205036@stu.sicau.edu.cn (C.Z.); hh820423@163.com (H.H.); 202101726@stu.sicau.edu.cn (S.Y.); 202101670@stu.sicau.edu.cn (Y.W.); huangzhi@sicau.edu.cn (Z.H.); 13920@sicau.edu.cn (Y.T.); 10650@sicau.edu.cn (H.L.); 2 Rice and Sorghum Research Institute, Sichuan Academy of Agricultural Sciences, Deyang 618000, China; lxmwsl@126.com; 3 Vegetable Germplasm Innovation and Variety Improvement Key Laboratory of Sichuan, Chengdu 610300, China; 4 School of Agriculture and Horticulture, Chengdu Agricultural College, Chengdu 611130, China; lhs8748@163.com

**Keywords:** malabar spinach, amaranth, sweet potato, phenolic compounds, antioxidants, antioxidant capacity

## Abstract

Malabar spinach (*Basella alba*), amaranth (*Amaranthus tricolor*), and sweet potato (*Ipomoea batatas*) are leafy vegetables found in Southwest China. The variation of chlorophyll, carotenoids, ascorbic acid, total flavonoids, phenolic compounds, and antioxidant capacity was studied in the leaves and stems of the three vegetables. The content of main health-promoting compounds and the antioxidant capacity in the leaves were higher than that in the stems, indicating that the leaves of the three vegetables possess greater nutritional value. The trend of total flavonoids in all three vegetables was similar to the trend of antioxidant capacity, suggesting that the total flavonoids may be the major antioxidants wihin these vegetables. Eight individual phenolic compounds were detected in three different vegetables. The most abundant levels of individual phenolic compounds in the leaves and stems of malabar spinach, amaranth, and sweet potato were 6′-*O*-feruloyl-d-sucrose (9.04 and 2.03 mg g^−1^ DW), hydroxyferulic acid (10.14 and 0.73 mg g^−1^ DW), and isorhamnetin-7-*O*-glucoside (34.93 and 6.76 mg g^−1^ DW), respectively. Sweet potato exhibited a higher total and individual phenolic compound content compared to malabar spinach and amaranth. Overall, the results demonstrate that the three leafy vegetables possess high nutritional value, and could be used not only for consumption but also in various other fields, including medicine and chemistry.

## 1. Introduction

**Figure 1:**
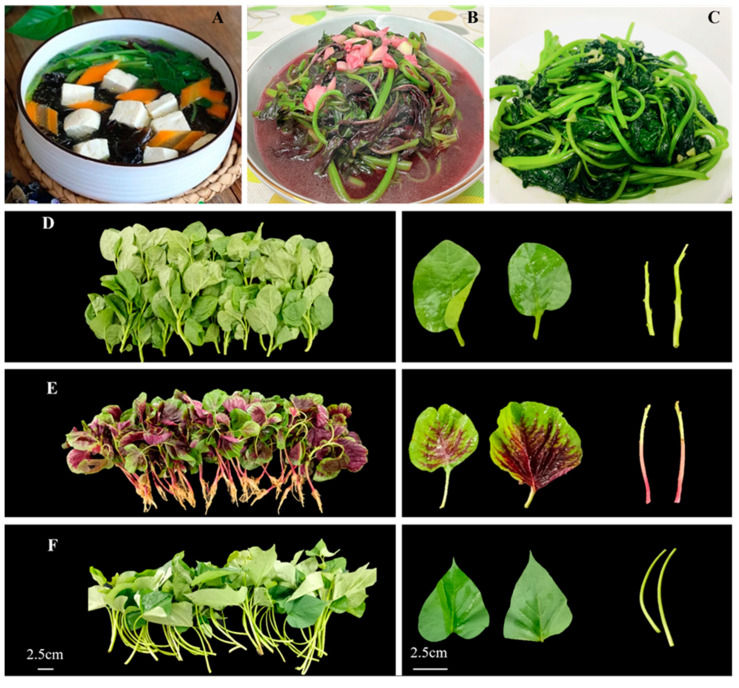
Popular dishes featuring malabar spinach (**A**), amaranth (**B**), and sweet potato (**C**), and the visual appearance of malabar spinach (**D**), amaranth (**E**), and sweet potato (**F**).

China has eight distinct vegetable growing areas, with the southwest region being the most prominent in terms of vegetable cultivation in the national medium-term genebank of vegetable germplasm resources [1]. Malabar spinach (*Basella alba*), amaranth (*Amaranthus tricolor*) and sweet potato (*Ipomoea batatas*) are three leafy vegetables popular in Southwest China due to their crisp mouthfeel and rich nutritional values. Malabar spinach is an annual twining herb, which belongs to the *Basellaceae* family. It originated in the tropical regions of Asia, and is also found in Africa and the Americas. Its edible parts include seedlings, tender shoots, and leaves [2,3]. In Southwest China, it is often used in soups to reduce their smooth flavor and enhance palatability among (Figure 1A). Amaranth is native to China, India, and Southeast Asia, among other places [4]. It is extensively cultivated in Southwest China from March to October [5]. Sweet potato is an important crop used for food, animal feed, and as an industrial raw material crop [6,7]. The main edible parts of sweet potato are tubers, but they are also consumed as an important leafy vegetable in Southwest China. Its leaves are also known as “Shao Jian” in Sichuan province. Due to farmers accidentally removing the tips of leaves for cooking during the production of feed, it was discovered that the leaves were palatable. Therefore, more and more people are using sweet potato leaves for cooking. Amaranth and sweet potato leaves are commonly stir-fried with garlic (Figure 1B,C) and pair well with rice, making them very popular among locals. The above three vegetables are widely cultivated as characteristic vegetables of Southwest China.

With the improvement of living conditions and the diversification of food options, there has been a gradual increase in the incidence of chronic diseases resulting from unhealthy dietary habits [8,9]. Numerous studies have shown that such diseases can be alleviated through the bioactive compounds in vegetable crops. Chlorophyll and carotenoids are color-forming pigments in plants and also provide health benefits [10]. Ascorbic acid and phenolic compounds are important antioxidant substances, which have potential anti-allergic, anti-inflammatory, and anti-bacterial properties [11]. There are more than 8000 kinds of phenolic compounds known in the plant kingdom, which are mainly composed of flavonoids, phenolic acids, tannins, etc., and each type is composed of many subclasses [12]. Studies have shown that phenolics can play an important role in the prevention of chronic diseases related to inflammation such as diabetes, cardiovascular diseases, etc. [13,14,15,16].

Malabar spinach, amaranth, and sweet potato are highly-sought-after leafy vegetables in the Southwest China, and have become characteristic vegetables of this region. However, research on the nutritional quality of these three vegetables is still limited. Additionally, different edible parts of the same plant can have variations in types and levels of nutrients [17,18,19]. In this study, we aimed to analyze the main phytochemicals and antioxidant capacities in the leaves and stems of the three leafy vegetables, and try to provide novel insights into the development and utilization of these three leafy vegetables in Southwest China.

## 2. Results

### 2.1. Chlorophyll

**Figure 2:**
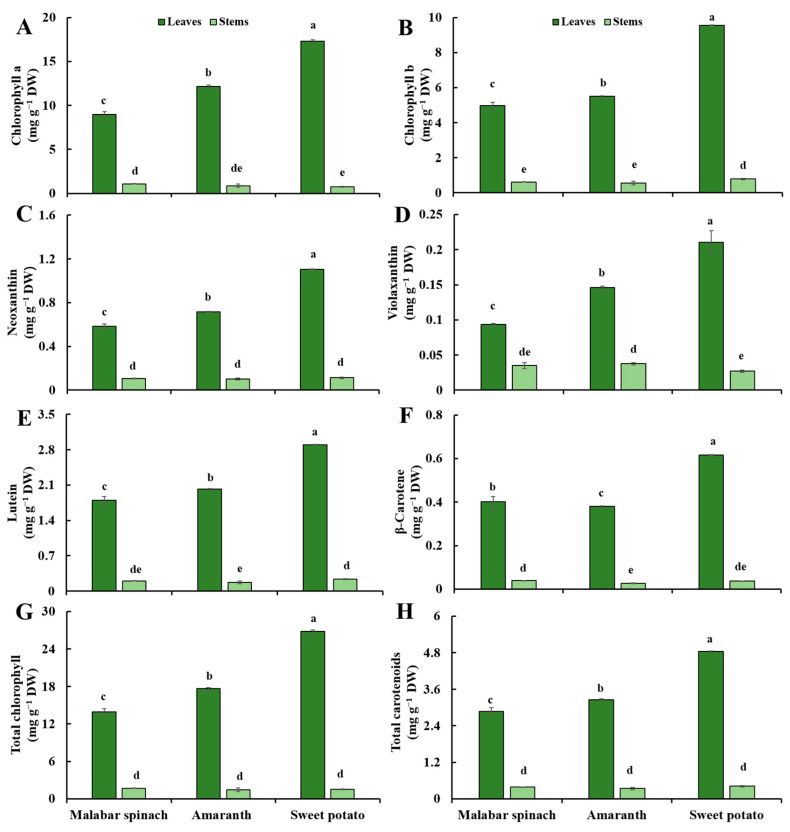
Pigments content in the leaves and stems among three species of vegetables. (**A**) chlorophyll a; (**B**) chlorophyll b; (**C**) neoxanthin; (**D**) violaxanthin; (**E**) lutein; (**F**) β-carotene; (**G**) total chlorophyll; (**H**) total carotenoids. Comparing six groups of samples, the same letter means no significant differences (*p* < 0.05) according to the LSD’s test.

Different vegetable species often exhibit variation in color. In general, the green color of malabar spinach and amaranth leaves was brighter and yellowish, while the sweet potato leaves tend to be dark green (Figure 1D–F). The chlorophyll content in the leaves of all three vegetable species was significantly higher than that in the stems. The chlorophyll content in the leaves was 8.1–23.8 times higher than that in the stems. Among these leafy vegetables, sweet potato exhibited the highest chlorophyll content in the leaves, followed by amaranth and malabar spinach. However, there was no significant difference in the concentrations of chlorophyll in the stems among these leafy vegetables (Figure 2A,B,G).

### 2.2. Carotenoids

Similar to chlorophyll, the carotenoid content in the leaves of all three leafy vegetables was higher compared to the stems. The content of carotenoids in the leaves was 2.7–16.9 times that of the stems. In the leaves, the content of carotenoids, except for β-carotene, was the highest in sweet potato, followed by amaranth, and malabar spinach had the lowest. The content of β-carotene was slightly different, with sweet potato having the highest content, followed by malabar spinach, and amaranth showing the lowest content. There was no significant difference in carotenoid concentration in the stems among the three vegetables (Figure 2C–F,H).

### 2.3. Ascorbic Acid

**Figure 3:**
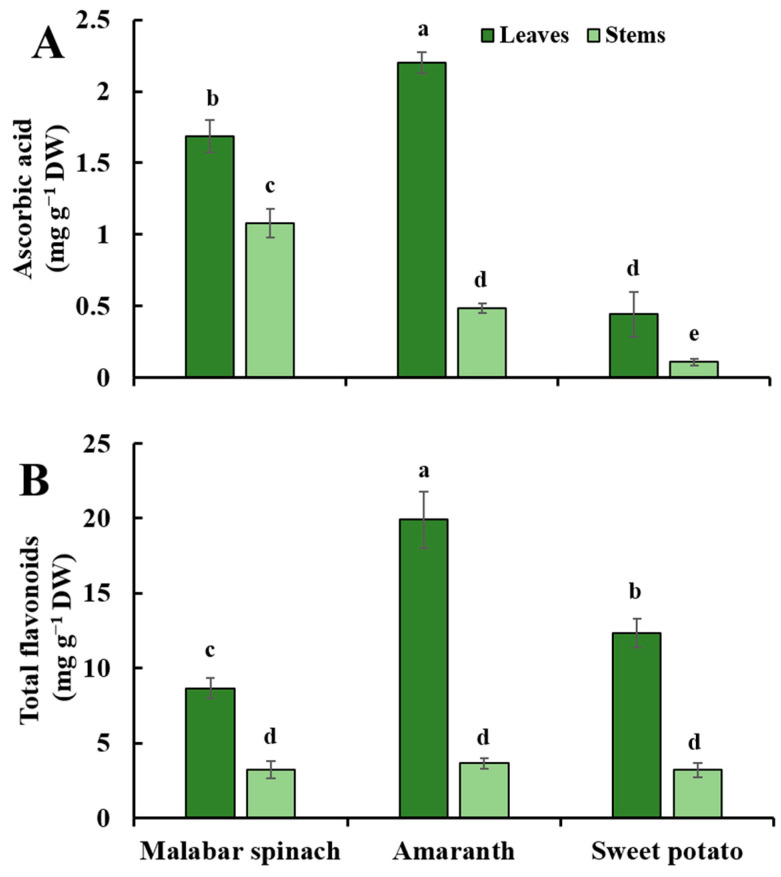
The ascorbic acid (**A**) and total flavonoids (**B**) in the leaves and stems among three species of vegetables. Comparing six groups of samples, the same letter means no significant differences (*p* < 0.05) according to the LSD’s test.

Among three leafy vegetables, the content of ascorbic acid in the leaves was significantly higher than that in the stems. The ratio of ascorbic acid content between leaves and stems was 1.6 to 4.6. In the leaves, amaranth had the highest content of ascorbic acid (2.20 mg·g^−1^ DW), followed by malabar spinach (1.69 mg·g^−1^ DW), and sweet potato had the lowest (0.44 mg·g^−1^ DW). In the stems, the content was the highest in malabar spinach (1.08 mg·g^−1^ DW) and the lowest in sweet potato (0.11 mg·g^−1^ DW), with a difference of 9.8 times (Figure 3A).

### 2.4. Total Flavonoids

The content of total flavonoids in the leaves was higher compared to the stems. The ratio of total flavonoid content between leaves and stems was the highest at 5.5 times in amaranth, and the lowest at 2.7 times in malabar spinach. Among the three species, the content of total flavonoids in the leaves was significantly different, which was the highest in amaranth (19.91 mg·g^−1^ DW) and the lowest in malabar spinach (8.68 mg·g^−1^ DW). However, there was no significant difference among the stems of the three species (Figure 3B).

### 2.5. Phenolic Compounds

**Figure 4:**
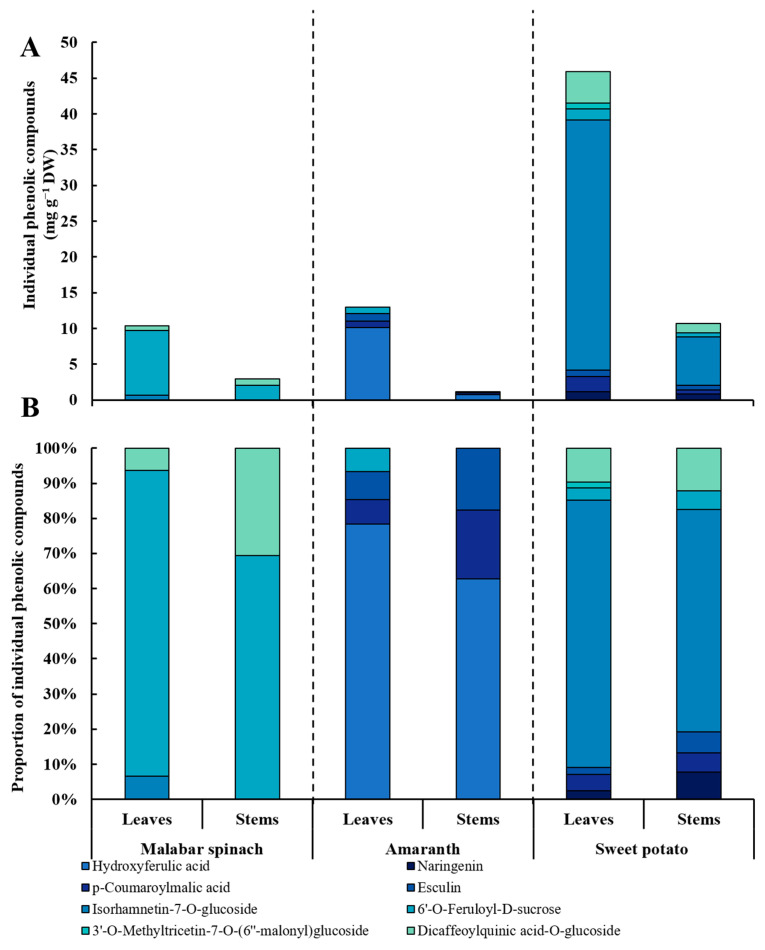
The stacked bar chart of individual phenolic compound content (**A**) and proportions (**B**) in the leaves and stems among three species of vegetables.

Eight kinds of phenolic compounds were detected in the three leafy vegetables, including four phenolic acids, three flavonoids, and one coumarin. Among the three different species, the leaves had a more abundant composition of phenolic compounds compared to the stems. For different species, only three and four phenolic compounds were detected in malabar spinach and amaranth, respectively. All kinds of phenolic compounds were detected in sweet potato except for hydroxyferulic acid. The 6′-*O*-feruloyl-d-sucrose was detected in all three leafy vegetables. Moreover, the content of total phenolic compounds was also the highest in sweet potato (Table 1 and Figure 4).

#### 2.5.1. Phenolic Acids

**Figure 5:**
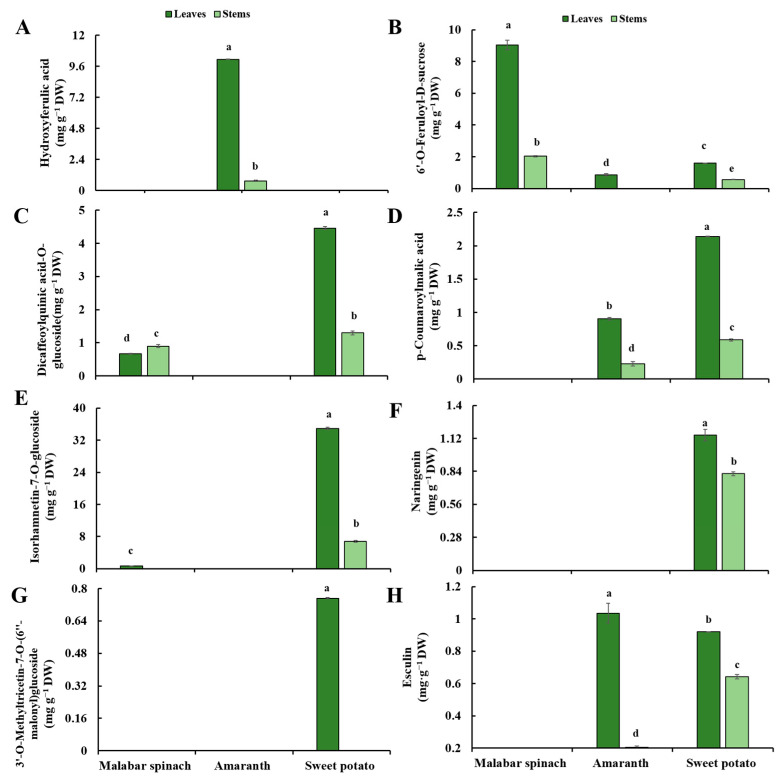
Individual phenolic compounds content in the leaves and stems among three species of vegetables. (**A**) hydroxyferulic acid; (**B**) 6′-*O*-Feruloyl-d-sucrose; (**C**) dicaffeoylquinic acid-*O*-glucoside; (**D**) *p*-coumaroylmalic acid; (**E**) isorhamnetin-7-*O*-glucoside; (**F**) naringenin; (**G**) 3′-*O*-methyltricetin-7-*O*-(6″-malonyl) glucoside; (**H**) esculin. Comparing six groups of samples, the same letter means no significant differences (*p* < 0.05) according to the LSD’s test.

The hydroxyferulic acid was only detected in amaranth, and the content was significantly higher in amaranth leaves (10.14 mg·g^−1^ DW) than in the stems (0.73 mg·g^−1^ DW) (Figure 5A). 6′-*O*-feruloyl-d-sucrose was detected in all samples except for the stems of amaranth. The content of 6′-*O*-feruloyl-d-sucrose in the leaves of malabar spinach (9.04 mg·g^−1^ DW) and sweet potato (1.60 mg·g^−1^ DW) was significantly higher than that in the stems (2.03 and 0.56 mg·g^−1^ DW, respectively). The highest content of 6′-*O*-feruloyl-d-sucrose in the leaves was observed in malabar spinach (9.04 mg·g^−1^ DW), followed by sweet potato (1.60 mg·g^−1^ DW), and the lowest in amaranth (0.87 mg·g^−1^ DW) (Figure 5B). Dicaffeoylquinic acid-*O*-glucoside was only detected in malabar spinach and sweet potato. The dicaffeoylquinic acid-*O*-glucoside content in the stems of malabar spinach was significantly higher than that in the leaves, while in sweet potato, the leaves had significantly higher level than the stems. The content of dicaffeoylquinic acid-*O*-glucoside was significantly higher in both the leaves and stems of sweet potato (4.46 and 1.30 mg·g^−1^ DW) compared to the corresponding parts of malabar spinach (0.67 and 0.89 mg·g^−1^ DW) (Figure 5C). The *p*-coumaroylmalic acid was detected in both amaranth and sweet potato, but not in malabar spinach. In both amaranth and sweet potato, the content of *p*-coumaroylmalic acid was significantly higher in the leaves compared to the stems. Furthermore, the content of *p*-coumaroylmalic acid was significantly higher in both the leaves and stems of sweet potato (2.14 and 0.59 mg·g^−1^ DW) than in the corresponding parts of amaranth (0.91 and 0.23 mg·g^−1^ DW), respectively (Figure 5D).

#### 2.5.2. Flavonoids

Isorhamnetin-7-*O*-glucoside was observed in the leaves of malabar spinach, as well as the leaves and stems of sweet potato. The highest isorhamnetin-7-*O*-glucoside content was detected in the leaves of sweet potato (34.93 mg·g^−1^ DW), and the lowest in the leaves of malabar spinach (0.68 mg·g^−1^ DW) (Figure 5E). In sweet potato, the content of isorhamnetin-7-*O*-glucoside in the leaves was significantly higher (34.93 mg·g^−1^ DW) than that in the stems (6.76 mg·g^−1^ DW). Naringenin was detected only in the sweet potato, with the content being significantly higher in the leaves (1.15 mg·g^−1^ DW) compared to the stems (0.82 mg·g^−1^ DW) (Figure 5F). Additionally, 3′-*O*-methyltricetin-7-*O*-(6″-malonyl) glucoside was observed in the leaves of sweet potato, with a content of 0.75 mg·g^−1^ DW (Figure 5G).

#### 2.5.3. Coumarins

Esculin was only detected in amaranth and sweet potato. The content of esculin was higher in the leaves compared to the stems in both of these species. The highest content of esculin was observed in the leaves of amaranth (1.04 mg·g^−1^ DW), while the lowest content was found in the stems of amaranth (0.21 mg·g^−1^ DW) (Figure 5H).

### 2.6. Antioxidant Capacity

**Figure 6:**
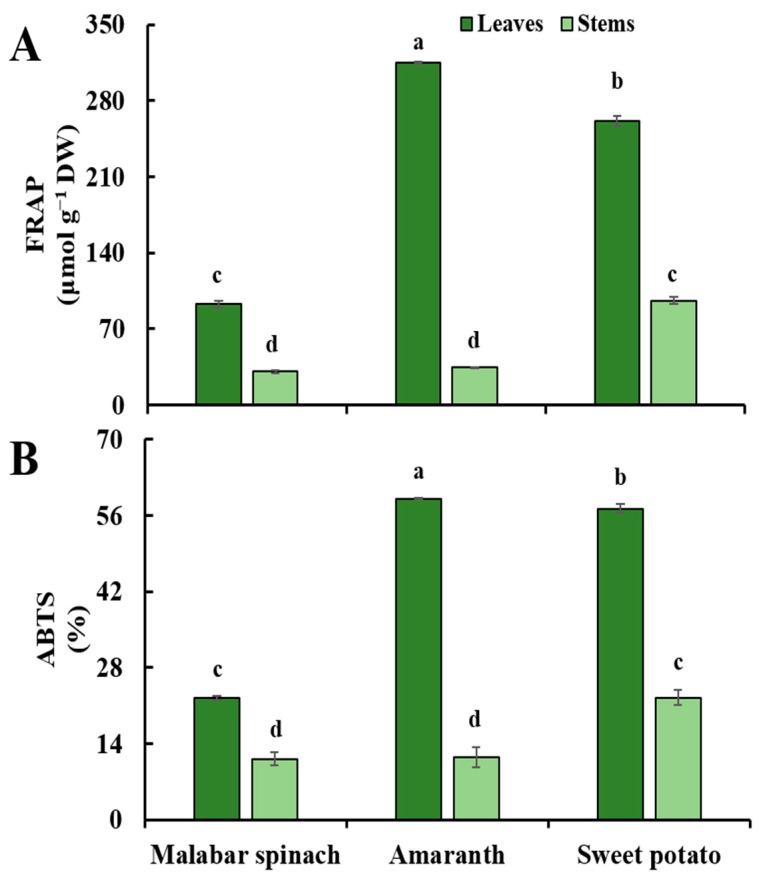
The antioxidant capacity in the leaves and stems among three species of vegetables by FRAP (**A**) and ABTS (**B**). Comparing six groups of samples, the same letter means no significant differences (*p* < 0.05) according to the LSD’s test.

The trends of antioxidant capacity, as measured by two methods (FRAP and ABTS), were similar. The antioxidant capacity in the leaves was higher compared to the stems of all three leafy vegetables. In the leaves, amaranth had the highest level of antioxidant capacity, followed by sweet potato, and malabar spinach had the lowest. In the stems, sweet potato had a higher antioxidant capacity compared to the other two species (Figure 6).

### 2.7. Principal Components Analysis (PCA)

**Figure 7:**
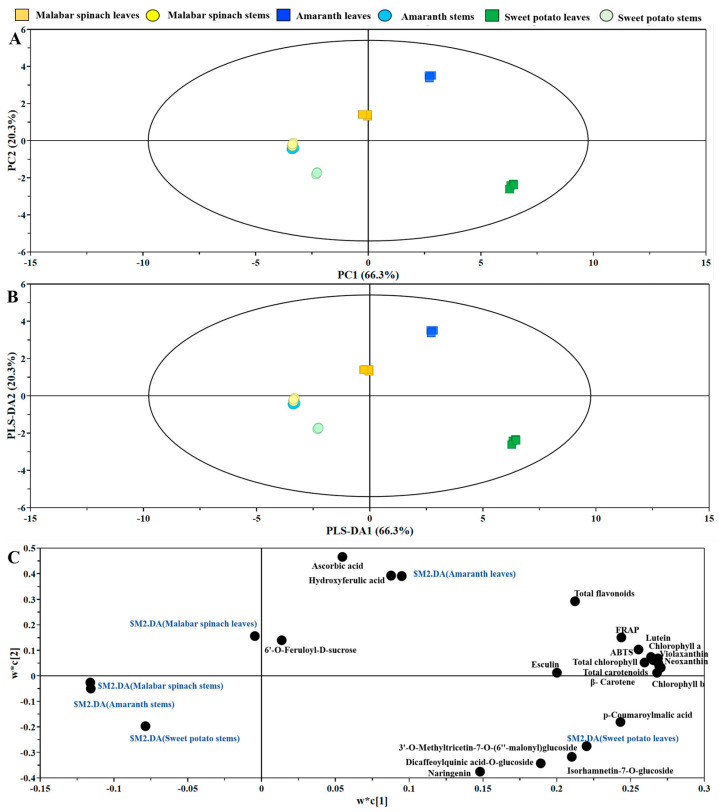
PCA analysis in the leaves and stems among three species of vegetables. (**A**) PCA Score plot; (**B**) PLS-DA Score plot; (**C**) loading plot.

A total of two principal components were obtained in the experiment. The first component (PC1) and second component (PC2) explained 66.3% and 20.3% of the variance, respectively. The leaves of malabar spinach, amaranth, and sweet potato could be discriminated by both PC1 and PC2, whereas the stems of the three vegetables were not clearly discriminated, especially the stems of malabar spinach and amaranth. In addition, the health-promoting compounds and antioxidant capacity of sweet potato were significantly separated from those in malabar spinach and amaranth. However, there was a partial overlap in the health-promoting compounds and antioxidant capacity between malabar spinach and amaranth, indicating a certain degree of similarity (Figure 7A). The results of the PLS-DA was consistent with that of the PCA (Figure 7B). According to the loading (Figure 7C) and VIP (Variable Importance in Projection) plots of the PLS-DA, the major contributors to the sweet potato leaves were *p*-coumaroylmalic acid.

### 2.8. Correlation Analysis

**Figure 8:**
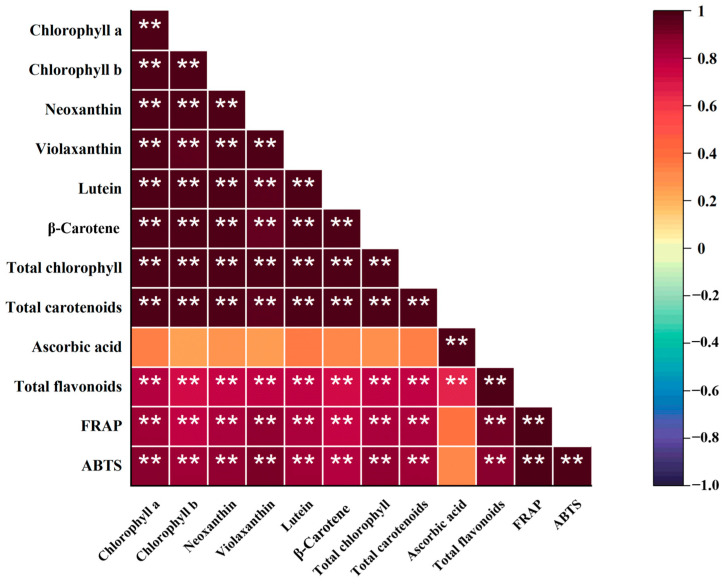
Correlation plot of the correlations between chlorophyll, carotenoids, ascorbic acid, total flavonoids, and antioxidant capacity of three species of vegetables. ** indicates the significance at 0.01 probability levels.

Chlorophyll, carotenoids, total flavonoids, and antioxidant capacity showed significant correlation, with correlation coefficients higher than 0.70. The correlation coefficient between total chlorophyll and neoxanthin was the highest, usually around 1.00. Ascorbic acid exhibited a significant correlation only with total flavonoids, with a correlation coefficient of 0.67 (Figure 8).

### 2.9. Variance Analysis

To assess the factors influencing the content of phytochemicals and the variation of antioxidant capacity, this experiment further analyzed the ratio of genetic variance component to phenotypic variance (Table 2). The variance ratio for all species of phytochemicals and antioxidant capacity, as well as the variance ratio for edible parts and the interaction (species × edible part) were significant at 0.01 levels. The ratios for the edible part variance were the highest among the species, edible part and interaction variances for chlorophyll, carotenoids, total flavonoids, and antioxidant capacity (FRAP and ABTS). The ratios for the species variance were the highest for ascorbic acid.

## 3. Discussion

In our study, we analyzed a variety of health-promoting compounds and the antioxidant capacity of malabar spinach, amaranth and sweet potato.

A large number of studies have confirmed remarkable differences in phytochemicals and antioxidant capacity among different organs and tissues of vegetables [20,21,22]. In our previous study, we found that the leaves of both purple flowering stalks and green flowering stalks had the higher content of health-promoting compounds, such as phenolics, compared to other edible parts [23]. The leaves of different variants of *Amaranthus* showed the highest content of total phenolics and FRAP values, while seeds and stalks had the lowest [24]. Similarly, in the case of taro (*Colocasia esculenta*), the health-promoting compound content was found to be higher in the leaves compared to other plant parts [25]. Generally, leaves possess higher nutritional value than petioles, roots, and other organs in many plant species [26]. A similar result was also obtained in our study. We found that the concentrations of chlorophyll, carotenoids, ascorbic acid, total flavonoids, and the antioxidant capacity were higher in the leaves than in the stems among the three vegetable species. The composition and content of phenolic compounds were also significantly higher in the leaves than those in the stems, except for dicaffeoylquinic acid-O-glucoside in malabar spinach. These observations may be attributed to the fact that leaves are the primary organs of photosynthesis in plants. Photosynthesis plays a crucial role in plant growth and productivity, providing the carbon skeleton needed for building plant structures and supporting growth [27,28]. Many products derived from photosynthesis are precursors to secondary metabolites (including chlorophyll, carotenoids, ascorbic acid, flavonoids, phenolic compounds) [29,30]. Therefore, as the main photosynthetic organ, leaves tend to contain more health-promoting compounds than stems.

Studies have demonstrated that vegetables (e.g., broccoli and cauliflower) have chemopreventive activity against chronic diseases because they have high levels of health-promoting compounds, including phenolic compounds and carotenoids [31]. Furthermore, phenolic compounds had a more significant contribution to the nutritional value. The leaves of *Moringa oleifera* Lam have attracted attention in food and medical fields because of their high phenolic content [32]. Similarly, *Amaranthus spinosus* has been proven to have high antioxidant and anti-inflammatory potential due to its abundance of phenolic bioactive molecules [33]. In line with these findings, our results show that among the three different vegetable species, leaves were more nutritious than stems. However, sweet potato leaves, which are often used as fodder, exhibit high nutritional value, particularly in terms of phenolic compounds. This discovery provides new insights into the utilization of the leaves among the three leafy vegetables.

For the health-promoting compounds in the leaves, there were also differences among the three tested vegetables. Previous studies have shown that chlorophyll, carotenoids, ascorbic acid, flavonoids and other health-promoting compounds have strong antioxidant capacity in vegetable crops [23,34,35,36,37,38], and phenolic compounds are also important antioxidants [39,40,41]. Our results suggested that for the content of chlorophyll and carotenoids, the trend was sweet potato > amaranth > malabar spinach. The content of ascorbic acid in different vegetable leaves showed the trend of amaranth > malabar spinach > sweet potato. The content of total flavonoids in different vegetable leaves showed the trend of amaranth > sweet potato > malabar spinach. The antioxidant activities measured by the two methods (FRAP and ABTS) were similar, the antioxidant capacity in the leaves followed the trend of amaranth > sweet potato > malabar spinach; which was similar to the trend of total flavonoids and different from the trend of chlorophyll, carotenoids, and ascorbic acid. The reasons for these findings may be as follows. Firstly, the antioxidant capacity is a result of the combined effect of all antioxidants present in the samples. There could be interactions between different antioxidants, which leads to variations in their levels. Secondly, different antioxidants contribute differently to the overall antioxidation capacity. In this study, it is possible that the total flavonoids made a major contribution to the antioxidation capacity. Chlorophyll and carotenoids, ascorbic acid and phenolic compounds might also have certain effect.

The composition and content of individual phenolic compounds in various vegetables was remarkably different. Phenolic compounds have been identified in many plants. For instance, hydroxycinnamic acid derivativesand other phenolic substances have been identified in papaya [42]. A total of 41 individual phenolic compounds were characterized and quantified in the pellicle and peeled kernels of six walnut cultivars [43]. Twenty-two phenolic substances were detected in three inbred varieties of *Lycium chinense* Miller leaves and stems [20]. In this experiment, the total content of eight phenolic compounds in leaves showed the trend of sweet potato > amaranth > malabar spinach, and the stems showed the trend of sweet potato > malabar spinach > amaranth. Among the eight individual phenolic compounds detected, seven of them were detected in sweet potato, four in amaranth, and three in malabar spinach. This is related to the species diversity of different vegetables, as well as planting, fertilization and management methods. The results showed that sweet potato had more kinds of individual phenolic compounds than the other two vegetables, and the content of these compounds was also higher in sweet potato, which provides an effective basis for the extraction and selection of phenolic compounds in the future.

Phenolic compounds have been widely recognized for their various properties, and are extensively used in medicine, chemical industry, and other fields [34,44]. For instance, caffeic acid [45], ferulic acid [46], quinic acid derivatives [47], and other phenolic compounds are well-known for their antioxidant and benefits to human beings. In our study, the predominant individual phenolic compounds in malabar spinach, amaranth, and sweet potato were 6′-*O*-feruloyl-d-sucrose, hydroxyferulic acid, and isorhamnetin-7-*O*-glucoside, respectively. The first two belong to phenolic acids, and the last one belongs to flavonoids. The content of decaffeoylquinic acid-*O*-glucoside, which belongs to phenolic acids, was relatively high in sweet potato and malabar spinach. Existing research on phenolic acids in pulse suggests their potential in the improvement of cardiovascular health [48,49]. Dicaffeoylquinic acids have proven to be promising therapeutic agents for managing diabetes and its complications (including cardiovascular disease) [50]. Isorhamnetin-7-*O*-glucoside has shown peroxynitrite and DPPH scavenging activities [51]. Free radicals such as DPPH play an important role in causing cardiovascular diseases such as atherosclerosis and heart failure, and are associated with numerous risk factors such as hypercholesterolemia, hypertension, and stress [52,53,54]. Therefore, isorhamnetin-7-*O*-glucoside has the potential to inhibit cardiovascular disease. Smoking is a significant risk factor for cardiovascular disease in China [55,56,57]. Researchers have demonstrated that a relatively high level of smoking was observed in Southwest China [58]. In Southwest China, local people favor the three vegetables because of their crisp mouthfeel and rich nutritional value. Phenolic compounds rich in these vegetables may reduce the risk of cardiovascular disease for local people. Thus, malabar spinach, amaranth, and sweet potato have potential in the prevention of cardiovascular diseases, offering new possibilities for their application in medicine, chemistry, and other fields.

## 4. Materials and Methods

### 4.1. Chemicals, Regents, and Instrumentation

Analytical-grade oxalic acid, potassium acetate, 2,2-azinobis (3-ethyl-benzothiazoline-6-sulfonic acid) (ABTS), and FRAP working solution were obtained from Sangon Biotech Co., Ltd. (Shanghai, China). Analytical-grade ethanol, acetone, petroleum, acetic acid, aluminum trichloride, and ferrous sulfate (FeSO_4_·7H_2_O) were purchased from Chengdu Kelong Chemical Co., Ltd. (Chengdu, China). The standards of chlorophylls (a and b), carotenoids (neo-xanthin, violaxanthin, lutein, and β-carotene), authentic ascorbic acid, and quercetin were obtained from Solarbio Science & Technology Co., Ltd. (Beijing, China). HPLC-grade isopropanol, acetonitrile, and methyl alcohol were purchased from Tedia Company, Inc. (Fairfield, OH, USA). The spectrophotometer was purchased from Shanghai Mapada Instruments Co., Ltd. in China. High-performance liquid chromatography (HPLC) analysis of ascorbic acid and individual phenolic compounds was carried out using a Shimadzu LC-10AT system equipped with a UV detector (Shimadzu Scientific Instruments Inc., Kyoto, Japan). The mass spectrometer (Agilent 1100 SL, Santa Clara, CA, USA) was equipped with dual electrospray ionization (ESI) source. The freeze-dryer FDU-2110 was purchased from Tokyo Physicochemical Equipment Co., Ltd. (EYELA) in Tokyo, Japan.

### 4.2. Plant Materials

Malabar spinach, amaranth, and sweet potato were planted at Chengdu experimental base at Sichuan Agricultural University. The herbarium specimen of malabar spinach was deposited in the Chongqing Institute of Drug Planting Herbarium under registry number IMC0084343, the herbarium specimen of amaranth was deposited in the Herbarium of Henan Agricultural University under registry number HEAC0016436, and the herbarium specimen of sweet potato was deposited in the Chongqing Institute of Traditional Chinese Medicine Herbarium under registry number SM716800748. Plant materials in good condition without disease and mechanical damage were sampled. There were 40 plants of each species of vegetable, divided into four replicates, with 10 plants per replicate. The samples were washed, air-dried at room temperature (20–25 °C) and relative humidity (60–70%) in dim-light for 30 min, separated into the leaves and the stems, and used for the determination of health-promoting phytochemicals. The samples were lyophilized in a freeze-dryer FDU-2110 (cold trap temperature: −80 °C) and then stored at −20 °C for analysis.

### 4.3. Determination of Chlorophyll and Carotenoids Content

Frozen powder (50 mg) was ground and extracted with 96% ethanol. After centrifugation, the supernatant was collected. Absorption was read at 665 nm and 649 nm with a spectrophotometer [59]. As for total carotenoids, frozen powder (50 mg) was extracted with a mixture of acetone and petroleum ether (1:1, *v*/*v*) and petroleum ether was used to ensure constant volume. Absorption was read at 451 nm with a spectrophotometer [59].

### 4.4. Determination of Ascorbic Acid

The sample powder was extracted with 1.0% oxalic acid, then centrifuged and separated at room temperature on a Waters Spherisorb C18 column (250 × 4.6 mm id; 5 μm particle size) using a solvent of 0.1% oxalic acid at a flow rate of 1.0 mL·min^−1^. Each sample was filtered and analyzed by HPLC. The amount of ascorbic acid was calculated from the absorbance values at 243 nm [59].

### 4.5. Determination of Total Flavonoids Content

After 24 h of darkness, the sample solution extracted with 50% ethanol was centrifuged and left for 40 min, then centrifuged. The supernatant was mixed with aluminum trichloride, potassium acetate, and distilled water. Absorbance was read at 415 nm using a spectrophotometer and total flavonoids content was determined using a standard calibration curve [60].

### 4.6. Determination of Individual Phenolic Compounds

Phenolic compounds were extracted and analyzed as previously described, with a minor modification [61]. Twenty milligrams of sample powder was weighed and extracted in 2 mL of 80% aqueous methanol solution. Then, the mixture was sonicated for 20 min after centrifugation for 7 min at 10,000 rpm, and the supernatant was obtained. The supernatant was filtered through a 0.22 μm nylon syringe filter for analysis. Phenolic compounds were carried out using HPLC, and chromatographic separation was performed at 30 °C on a 250 mm × 4.6 mm, 5 μm Symmery C18 column (Agilent, Santa Clara, CA, USA). The mobile phases consisted of eluent A (methanol/acetic acid/water, 10:2:88, *v*/*v*/*v*) and eluent B (methanol/acetic acid/water, 88:2:10, *v*/*v*/*v*), using a gradient program as follows: 0~15 min, 10% B~30% B; 15~26 min, 30% B; 26~28 min, 90% B; 28~35 min, 10% B. The flow rate was 1 mL·min^−1^, the injection volume was 5 μL, and the absorbance was detected at 340 nm. HPLC with mass spectrometry (HPLC-MS) system was used to identify phenolic compounds. The MS was set as follows: Mass spectral data was obtained using ESI in positive and negative ionization modes over the range of *m*/*z* 100~1000. The N_2_ temperature was 350 °C, and the dry gas flow was set to 9 mL·min^−1^. The capillary voltage, fragmentor voltage, skimmer voltage, and octopole voltage were 4000, 175, 65, and 750 V, respectively. The amount of each phenolic compound was shown in equal amounts of rutin.

### 4.7. Ferric Reducing Antioxidant Power (FRAP)

The extracted samples were added to the FRAP working solution incubated at 37 °C. After incubation for 10 min, the absorbance was recorded at 593 nm using a spectrophotometer. FRAP values were calculated according to FeSO_4_·7H_2_O standard curves and expressed as μmol·g^−1^ dry weight [62].

### 4.8. 2,2-Azinobis (3-Ethyl-benzothiazoline-6-sulfonic Acid) (ABTS) Assay

For each extraction sample, 300 μL of equal solution was added to 3 mL of ABTS^+^ solution. The absorbance was measured spectrophotometrically at 734 nm after 2 h. The percentage inhibition was calculated according to the formula: % inhibition = [(A_control_ − A_sample_)/A_control_] × 100% [63].

### 4.9. Statistical Analysis

All trials were performed in quadruplicate. Statistical analysis was performed using the SPSS package, version 18 (SPSS Inc., Chicago, IL, USA). Two-way analysis was used for variance. The least significant differences (LSD) test was used for comparison, and the significance level was 0.05. Principal component analysis (PCA) was performed in SIMCA-P 14.1 (Umetrics, Umeå, Sweden) with unit-variance (UV) scaling to decipher the relationships among samples. The correlation results were visualized using Origin 2022 (OriginLab Corporation, Wellesley, MA, USA) [64].

## 5. Limitations and Future Implications

In this study, we mainly focused on evaluating the health-promoting compounds of three leafy vegetables, and the research on antioxidant capacity was not comprehensive enough. In the future, additional methods could be employed to measure antioxidant capacity, and more in-depth research on antioxidant enzymes (superoxide dismutase, glutathione peroxidase, etc.) could be conducted. This will contribute to a more comprehensive understanding of the vegetables’ antioxidant potential. Moreover, it is suggested that future research explores the nutritional composition of different vegetables from various perspectives to enable more diversified utilization in fields such as medicine and food processing. We hope to draw more attention to health promoting compounds present in these three leafy vegetables through this study, and provide new insights for the application of vegetables in various fields.

## 6. Conclusions

In this study, we analyzed the main health-promoting compounds and antioxidant capacities in the leaves and stems of malabar spinach, amaranth, and sweet potato. The results show that the leaves of these vegetables contain significantly higher levels of phenolic compounds, chlorophyll, carotenoids, ascorbic acid, and total flavonoids compared to the stems. This indicates that the leaves of these three vegetable species possess greater nutritional value. Additionally, we observed a similar trend between flavonoid content and antioxidant capacity, suggesting that total flavonoids may contribute significantly to the overall antioxidant activity in these vegetables. Furthermore, sweet potato exhibited a higher variety and content of individual phenolic compounds compared to malabar spinach and amaranth, indicating its potential as a raw material for extracting phenolic substances. The main individual phenolic compounds identified in malabar spinach, amaranth, and sweet potato were 6′-*O*-feruloyl-d-sucrose, hydroxyferulic acid, and isorhamnetin-7-*O*-glucoside, respectively. Moreover, the phenolic compounds abundant in these vegetables may offer benefits in reducing the risk of cardiovascular disease, expanding their potential applications beyond mere consumption to fields such as medicine, chemistry, and more.

## Figures and Tables

**Table 1:** The information obtained in the ESI mass spectrometer for each phenolic analyzed.

Classified	Phenolic	*M_W_*	Negative Ionization Model MS (*m*/*z)*	Positive Ionization Model MS (*m*/*z)*
Phenolic acids	Hydroxyferulic acid	209.9	208.9 [M − H]^−^	249 [M + K]^+^
Phenolic acids	*p*-Coumaroylmalic acid	280.2	279.2 [M − H]^−^/ 393.2 [M + CF_3_COO]^−^	298.1 [M + NH_4_]^+^
Phenolic acids	6′-*O*-Feruloyl-d-sucrose	518	517 [M − H]^−^/ 555 [M + Cl]^−^	519 [M + H]^+^
Phenolic acids	Dicaffeoylquinic acid-*O*-glucoside	678.4	713.4 [M + Cl]^−^/ 737.4 [M + CH_3_COO]^−^	701.4 [M + Na]^+^/ 679.4 [M + H]^+^
Flavonoids (Flavonols)	Isorhamnetin-7-*O*-glucoside	478	515 [M + Cl]^−^/ 537 [M + CH_3_COO]^−^	538.9 [M + Na + CH_3_CN]^+^/ 499 [M + Na]^+^
Flavonoids (Flvanones)	Naringenin	272	385 [M + CF_3_COO]^−^	294.7 [M + Na]^+^/ 272.7 [M + H]^+^
Flavonoids (Flavones)	3′-*O*-Methyltricetin-7-*O*-(6″-malnyl) glucoside	564.1	563.1 [M − H]^−^/ 599.1 [M + Cl]^−^	585 [M + Na]^+^
Coumarins	Esculin	339.9	384.9 [M + HCOO]^−^	362.2 [M + Na]^+^/ 340.2 [M + H]^+^

**Table 2:** Estimated proportions of variance components for chlorophyll, carotenoids, ascorbic acid, total flavonoids, and antioxidant capacity among the three species of vegetable.

Parameter	V_S_/V_P_	V_E_/V_P_	V_SE_/V_P_
Chlorophyll a	0.065 **	0.857 **	0.077 **
Chlorophyll b	0.102 **	0.812 **	0.086 **
Neoxanthin	0.087 **	0.830 **	0.082 **
Violaxanthin	0.107 **	0.743 **	0.142 **
Lutein	0.053 **	0.900 **	0.046 **
β-Carotene	0.057 **	0.890 **	0.052 **
Total chlorophyll	0.076 **	0.845 **	0.079 **
Total carotenoids	0.061 **	0.882 **	0.056 **
Ascorbic acid	0.472 **	0.355 **	0.161 **
Total flavonoids	0.156 **	0.695 **	0.131 **
FRAP	0.243 **	0.592 **	0.164 **
ABTS	0.249 **	0.607 **	0.142 **

V_S_/V_P_: ratio of species variance to phenotypic variance; V_E_/V_P_: ratio of edible part variance to phenotypic variance; V_SE_/V_P_: ratio of species × edible part interaction variance to phenotypic variance. ** indicates the significance at 0.01 probability levels in the same column.

## Data Availability

All data supporting the findings of this study are available within the paper and within its Supplementary Materials published online.

## Supplementary Materials

The following supporting information can be downloaded at: https://www.mdpi.com/article/10.3390/molecules28124780/s1, Table S1: Correlation coefficients between chlorophyll, carotenoids, ascorbic acid, total flavonoids, and antioxidant capacity of three species of vegetables.
