# Supplementary material for: Variation in the Main Health-Promoting Compounds and Antioxidant Capacity of Three Leafy Vegetables in Southwest China
Source: Molecules. 2023 Jun 15;28(12):4780. doi: 10.3390/molecules28124780 (PMC10303137; doi:10.3390/molecules28124780)
Supplement: Supplementary file 1 [file molecules-28-04780-s001.zip › molecules-2451638-supplementary.pdf]

Supplemental Table S1

Correlation coefficients between chlorophyll, carotenoids, ascorbic acid, total flavonoids, and antioxidant capacity of three species of vegetables.

	Chlorophyll a	Chlorophyll b	Neoxanthin	Violaxanthin	Lutein	β-Carotene	Total chlorophyll	Total carotenoids	Ascorbic acid	Flavonoid	FRAP	ABTS
Chlorophyll a	1											
Chlorophyll b	0.990888	1										
Neoxanthin	0.997499	0.99773	1									
Violaxanthin	0.986469	0.977356	0.985329	1								
Lutein	0.994845	0.99136	0.994316	0.967744	1							
β- Carotene	0.984469	0.990891	0.988435	0.9524	0.995954	1						
Total chlorophyll	0.998936	0.996045	0.999629	0.985377	0.995697	0.988692	1					
Total carotenoids	0.996277	0.994674	0.996972	0.97329	0.999529	0.995695	0.997776	1				
Ascorbic acid	0.351841	0.251541	0.293668	0.274817	0.366289	0.337558	0.318225	0.344069	1			
Flavonoid	0.810212	0.729272	0.770901	0.786431	0.7889	0.737577	0.784166	0.780163	0.666437	1		
FRAP	0.854918	0.798794	0.831807	0.863734	0.820983	0.768307	0.837461	0.820192	0.398035	0.925959	1	
ABTS	0.890941	0.847751	0.874691	0.904133	0.857641	0.811386	0.877986	0.859288	0.328816	0.895319	0.992205	1
